# lncRNA-SOX2OT promotes hepatocellular carcinoma invasion and metastasis through miR-122-5p-mediated activation of PKM2

**DOI:** 10.1038/s41389-020-0242-z

**Published:** 2020-05-28

**Authors:** Yingjian Liang, Dandan Zhang, Tongsen Zheng, Guangchao Yang, Jiabei Wang, Fanzheng Meng, Yao Liu, Guoli Zhang, Linhan Zhang, Jihua Han, Peng Hui, Zhengliang Chen, Yu Liu, Mingyu Wang, Hongchi Jiang, Lianxin Liu

**Affiliations:** 1 Key Laboratory of Hepatosplenic Surgery, Ministry of Education, Department of General Surgery, The First Affiliated Hospital of Harbin Medical University, Harbin, Heilongjiang 150001 China; 2 Department of Gynaecology and Obstetrics, The First Affiliated Hospital of Harbin Medical University, Harbin, Heilongjiang 150001 China; 3 Department of GI Oncology, Harbin Medical University Cancer Hospital, Harbin, China; 4 The First Affiliated Hospital of USTC, Division of Life Sciences and Medicine, University of Science and Technology of China, Hefei, China; 5 Outpatient Department, The First Affiliated Hospital of Harbin Medical University, Harbin, Heilongjiang 150001 China; 6 Department of Radiology, The First Affiliated Hospital of Harbin Medical University, Harbin, Heilongjiang 150001 China; 7 Department of Head and Neck Surgery, The Third Affiliated Hospital of Harbin Medical University, Harbin, China

**Keywords:** Cancer metabolism, Liver cancer, Cancer metabolism, Liver cancer

## Abstract

Tumor cells primarily utilize aerobic glycolysis for energy production, a phenomenon known as the Warburg effect, but the involvement of Warburg effect in liver cancer cell metastasis is not well understood. In present study, our results indicate a positive correlation between glucose metabolism level and metastatic potential of hepatocellular carcinoma (HCC). We also observed that a long noncoding RNA-SOX2OT (lncRNA-SOX2OT) can not only increase the metastatic potential of HCC but also promote a pyruvate kinase M2 (PKM2)-mediated activation of glucose metabolism. Inhibition of PKM2 in HCC cells greatly compromises lncRNA-SOX2OT in promoting Warburg effect and metastasis. Furthermore, miR-122-5p was found being a direct target of lncRNA-SOX2OT in regulating PKM2 expression. Thus, our findings reveal that lncRNA-SOX2OT, a regulator of PKM2, could predispose HCC patients to metastases and may serve as a candidate for metastatic prediction and therapies in HCC patients.

## Introduction

Hepatocellular carcinoma (HCC) is a common and aggressive human malignancy^1,2^, with a dismal outcome that is largely due to metastasis and postsurgical recurrence. Unfortunately, the 5-year survival rate of HCC patients remains poor^3^. Novel molecular mechanisms that can help determine how HCC metastasizes and improve treatment are still urgently needed.

Cancer metabolic reprogramming has been recognized as one of the ten cancer hallmarks^4^. Cancer cells favor the shift to glycolytic metabolism, referred to as the “Warburg effect”, a phenomenon whereby cancer cells rely mainly on aerobic glycolysis to generate adenosine triphosphate (ATP) even in the presence of O_2_^5–8^. As a result of oxidative stress tolerance, the cancer cells are therefore more likely to evade anoikis and even acquire metastatic potential. Essentially, the Warburg effect promotes the growth and metastasis of tumors^9–12^.

Since liver is responsible for most of the task of energy metabolism in the human body, abnormal expression of some key enzyme was found of relationship with the tumorigenesis of HCC. The results of Kwee et al.^13^ indicated biological and prognostic significance of hexokinase (HK2) and choline kinase alpha (CKA) expression in HCC. Fan et al.^14^ provided evidence that pyruvate kinase isozyme type M2 (PKM2) can regulate β-catenin-TCF/LEF-1 transcriptional activity and associated epithelial-to-mesenchymal transition (EMT) in HCC cell lines. Amann et al.^15^ showed that high expression of the glucose transport protein (Glut1) is positively correlated with the malignancy of HCC. These results support the notion that metabolic alterations may contribute to the metastatic phenotype of HCC. However, the possible mechanism is unclear.

The human transcriptome comprises not only large numbers of protein-coding messenger RNAs (mRNAs), but also a large set of nonprotein-coding transcripts that have structural, regulatory, or unknown functions^16^. Although studies of small noncoding RNAs have dominated the field of RNA biology in recent years^17^, long noncoding RNAs (lncRNAs) have been shown to play significant regulatory roles in development^18^, differentiation^19^, and carcinogenesis^20^. Yuan et al.^21^ found that transforming growth factor-β (TGF-b) can activate lncRNA-ATB and promotes the colonization of disseminated HCC cells. Moreover, Huang et al.^22^ reported that lncRNA-Dreh could inhibit HCC metastasis by modifying the expression and reorganization of vimentin. Given that the contribution of lncRNAs to the regulation of “Warburg Effect” in HCC has never been discussed before, we wondered whether lncRNAs could affect the level of carbohydrate metabolism, thereby regulating HCC metastasis.

In the present study, our results indicate that lncRNAs-SOX2OT which can regulate glycolysis is dysregulated in patients with metastatic HCC. We further investigated the biological function of a lncRNA-SOX2OT, in vivo and in vitro, and found that it plays an important role in liver cancer metastasis.

## Results

### The SUVmax level of tumor exhibited significant correlation with HCC metastasis

**Fig. 1:**
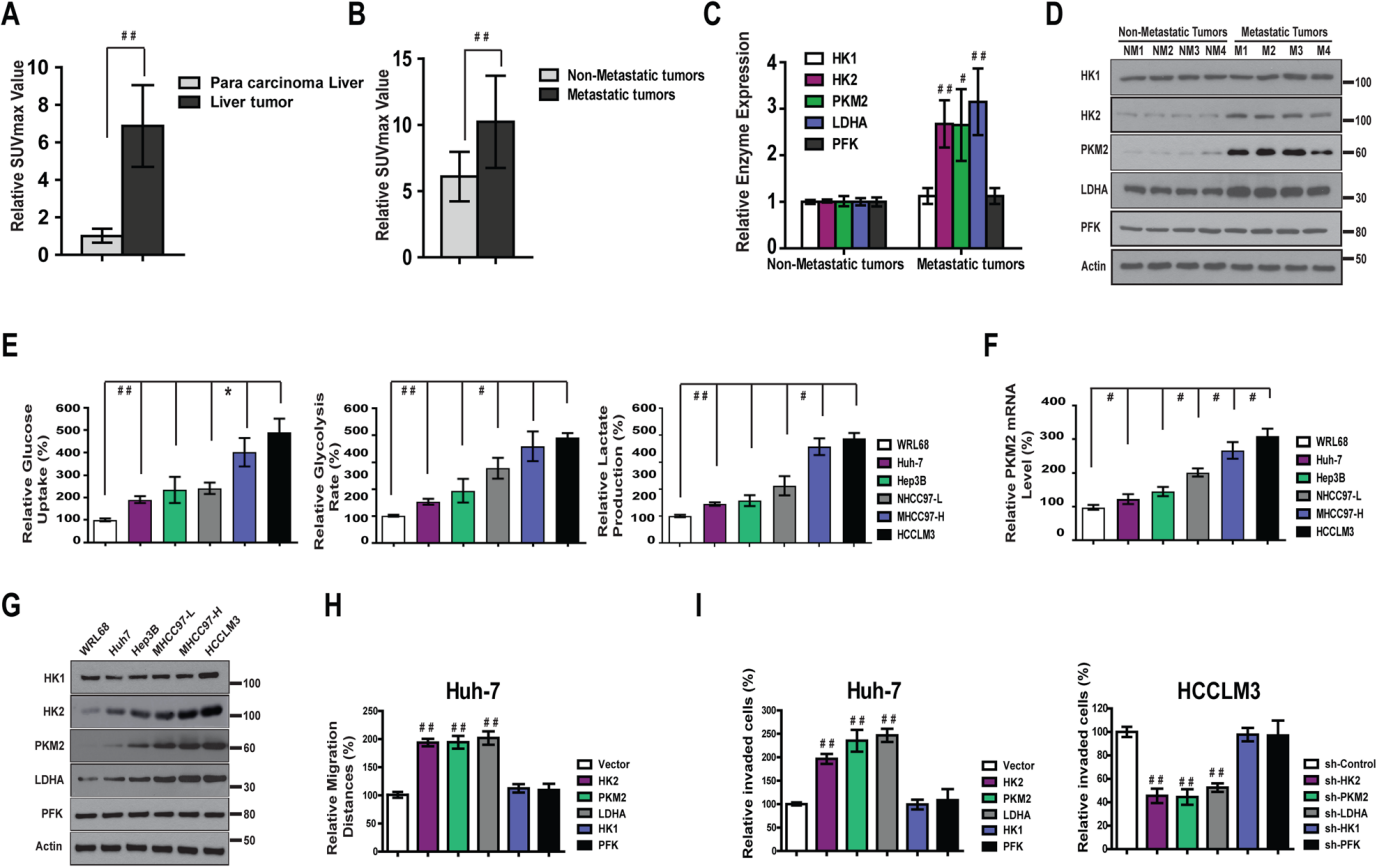
The level of metabolism is positively correlate with metastatic potential of HCC. **a** The levels of Suvmax (*n* = 121) value in were significantly higher in liver tumor than in para carcinoma tissue. **b** Suvmax levels were significantly higher in metastatic liver tissue (*n* = 32) than in non-metastatic HCC tissue (*n* = 84). **c**, **d** The mRNA and protein expressions of HK2, PKM2, and LDHA were much higher in HCC tumors that had more metastatic potential. **e** Relative glucose uptake, the rate of glycolysis and lactate production were higher in five HCC cell lines compared with normal liver cell line WRL68. **f** Relative PKM2 mRNA level was higher in five HCC cell lines compared with normal liver cell line WRL68. **g** Protein levels of HK2, PKM2, and LDHA were much higher in five HCC cell lines compared with normal liver cell line WRL68. **h** Overexpression of HK2, PKM2, and LDHA markedly increased wound healing capacity in Huh-7 cells. **i** Overexpression of HK2, PKM2, and LDHA markedly increased Matrigel invasion in Huh-7 cells. On the contrary, knockdown of HK2, PKM2, and LDHA block Matrigel invasion in HCCLM3 cells.

One hundred twenty-one HCC patients who underwent 18F-FDG PET scans for the detection of extrahepatic metastasis between January 2005 and December 2008 were identified at the First Affiliated Hospital of Harbin Medical University, China. The value of SUVmax was analyzed to evaluate the level of carbohydrate metabolism. The expression level of SUVmax was significantly upregulated in HCC tissue versus adjacent non-tumor liver tissue (*P* < 0.001) (Fig. 1a and Fig. S1A). Furthermore, SUVmax levels were significantly higher in metastatic liver tissues than in nonmetastatic HCC tissues (Fig. 1b and Fig. S1A), indicating that liver cancer with a high level of glucose metabolism may have greater metastatic potential. This notion is consistent with the reprogrammed energy metabolism characteristic of cancer cells which termed “aerobic glycolysis”^8^.

To determine the glycolytic level in HCC samples with different metastatic potential, we first examined the expression of some enzymes involved in glycolysis. The mRNA levels of Hexokinase 1 (HK1), HK2, PKM2, lactate dehydrogenase A (LDHA), and phosphofructokinase (PFK) were evaluated using real-time polymerase chain reaction (PCR). The results indicated that the expression of HK2, PKM2, and LDHA were much higher in HCC tumors that had more metastatic potential (Fig. 1c). Consistent with the mRNA expression, the protein levels of HK2, PKM2, and LDHA were elevated in HCC samples that had higher metastatic potential (Fig. 1d). However, no significant change in HK1 and PFK level was observed. These findings indicated that the glycolytic level of HCC might be correlated with metastatic features.

### The level of glycolysis exhibited significant correlation with metastatic potential of HCC cells

To ascertain the correlation between glycolytic level and metastatic potential in HCC cells, the level of glycolysis^23,24^ was evaluated in five HCC cell lines (Hep3B, Huh-7, MHCC97-L, MHCC97-H, and HCCLM3) and one normal liver cell line (WRL68). We observed that the level of glucose uptake and the rate of glycolysis and lactate production (Fig. 1e) in highly metastatic cell lines (MHCC97-H and HCCLM3) were significantly higher than in the HCC cell lines with lower metastatic potential (Hep3B, MHCC97-L, and Huh-7) and the normal liver cell line (WRL68). These data indicate that the level of glycolysis is elevated in HCC cell lines, and its upregulation is positively correlated with the metastasis potential of HCC cells.

The expression of HK1, HK2, PKM2, LDHA, and PFK, which are crucial in the glycolytic pathway, were also evaluated. The results demonstrated significant increases in HK2, PKM2, and LDHA mRNA levels in MHCC97-H and HCCLM3 cells, which have higher metastatic potential (Fig. 1f and Fig. S1B). Similarly, when the protein level was evaluated, the results (Fig. 1g) indicated elevated expression of HK2, PKM2, and LDHA in MHCC97-H and HCCLM3 cells.

To further investigate the correlation between HK2, PKM2, LDHA, and HCC cell metastasis, we performed a wound-healing assay and Transwell assay using Huh7 and MHCC97-H cells. Overexpression of HK2, PKM2, and LDHA markedly increased wound healing and Matrigel invasion capacity in Huh-7 cells (Fig. 1h, i and Fig. S1C, S1D). Conversely, knocking down HK2, PKM2, and LDHA expression significantly suppressed Matrigel invasion capacity in HCCLM3 cells (Fig. 1i and Fig. S1D).

### Aberrant expression of lncRNA-SOX2OT is associated with metastatic potential of HCC

**Fig. 2:**
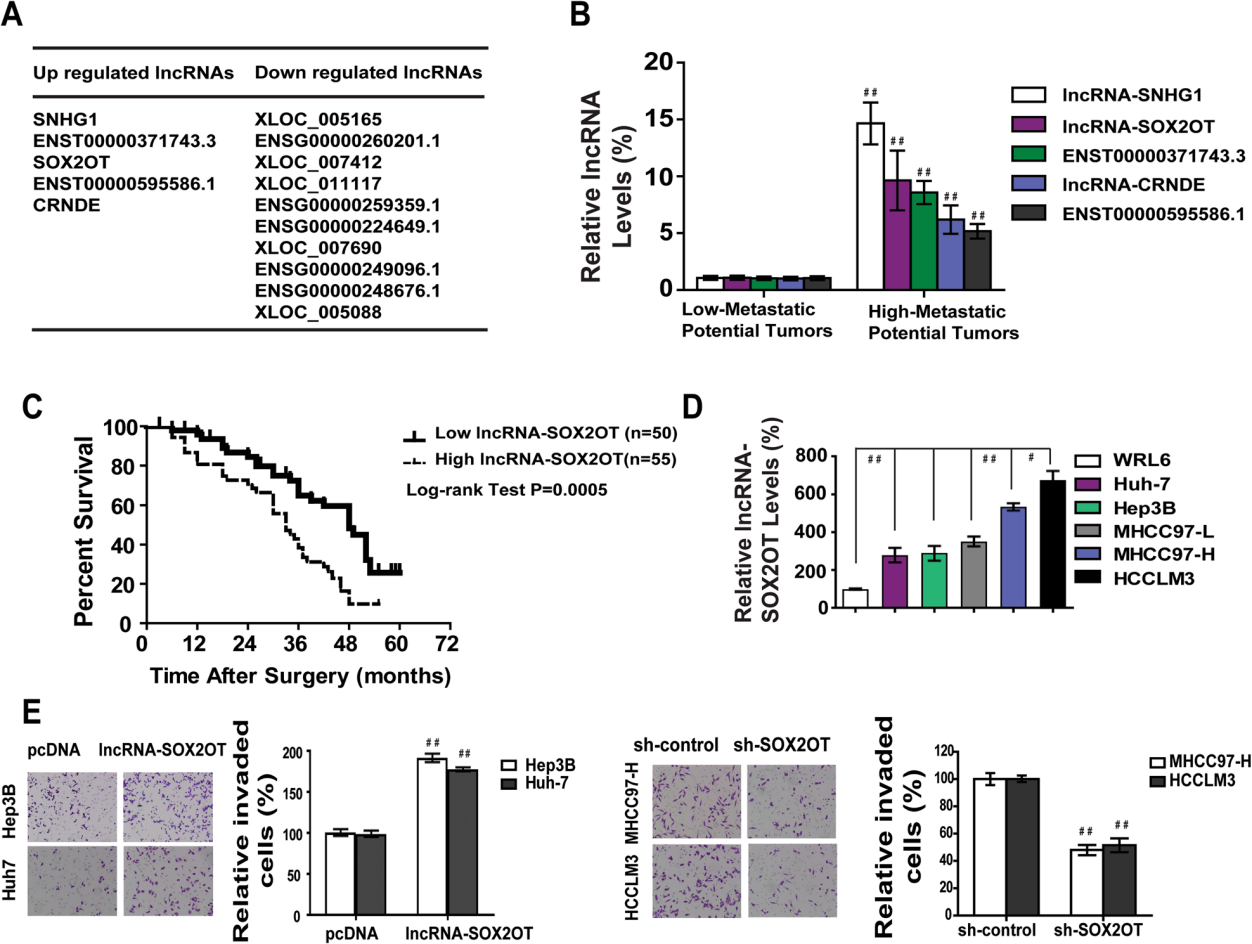
lncRNA-SOX2OT is up-regulated in HCC of higher metastatic potential. **a** Fifteen candidate lncRNAs were selected from lncRNA expression profile in ten pairs of HCC tissue having different metastatic potential. **b** Much higher expressions of five lncRNAs would be observed in tumor tissue with high metastatic potential. **c** Patients with high level of lncRNA-SOX2OT indicates dismal survival after surgery. **d** Relative lncRNA-SOX2OT expressions were much higher in five HCC cell lines compared with normal liver cell line WRL68. **e** Overexpression of lncRNA-SOX2OT promoted metastasis of Hep3B and Huh-7 cells. Knockdown of lncRNA-SOX2OT blocked metastasis of MHCC97-H and HCCLM3 cells.

lncRNAs are reportedly responsible for various biological processes in HCC^25^. To determine the overall impact of lncRNAs on HCC metastasis, we used microarray analysis to analyze the expression profile of lncRNAs in ten pairs of human HCC samples with different metastatic outcomes. We set a threshold as fold change >1.5, and found that there were five upregulated lncRNAs and ten downregulated lncRNAs in liver tumors that had high metastatic potential (Fig. 2a and Fig. S2A). This finding indicates that the lncRNA expression profiles are different between the two groups.

To validate the microarray analysis results, we selected five lncRNAs from the differentially expressed lncRNAs with fold change >2.5 and checked their expression by real-time PCR in a panel of 105 paired HCC/non-tumor tissue specimens with different metastatic potential (Fig. 2b). The results were consistent with the microarray results. To explore whether these lncRNAs could be important factors in determining clinical outcomes of HCC patients, we examined the correlation between the expression of the 5 tested lncRNAs and the prognosis of the 105 pairs of HCC samples. Patient characteristics are listed in Supplementary Table 1. The data indicated that patients with high lncRNA-SOX2OT expression in HCC had significantly worse prognosis than did those with lower expression (Fig. 2c). These findings suggest an important role of lncRNA-SOX2OT in hepatocarcinogenesis and metastasis.

To further analyze the association of lncRNA-SOX2OT with HCC metastasis, we measured lncRNA-SOX2OT level in a panel of HCC cell lines with different metastatic potential. We observed that lncRNA-SOX2OT expression in HCC cell lines with high metastatic potential (HCCLM3 and MHCC97-H) was significantly higher than expression in the HCC cell lines with low metastatic potential (Hep3B and Huh-7; Fig. 2d). These data indicated that elevated expression of lncRNA-SOX2OT was positively correlated with the metastatic potential of HCC cells.

Next, lncRNA-SOX2OT was stably upregulated or knocked down in Huh-7 and HCCLM3 cell lines (Fig. S2B). The results of Wound-healing assay and Matrigel migration assays showed that the mobility and migration of Huh-7 and Hep3B cells was significantly increased after transfection with lncRNA-SOX2OT. Knockdown of lncRNA-SOX2OT blocked wound healing and migration of MHCC97-H and HCCLM3 cells (Fig. S2C, E). Taken together, these data suggest that the expression of lncRNA-SOX2OT is positively correlated with the metastatic potential of HCC cells.

### Effects of lncRNA-SOX2OT on in vivo tumor metastasis of HCC

**Fig. 3:**
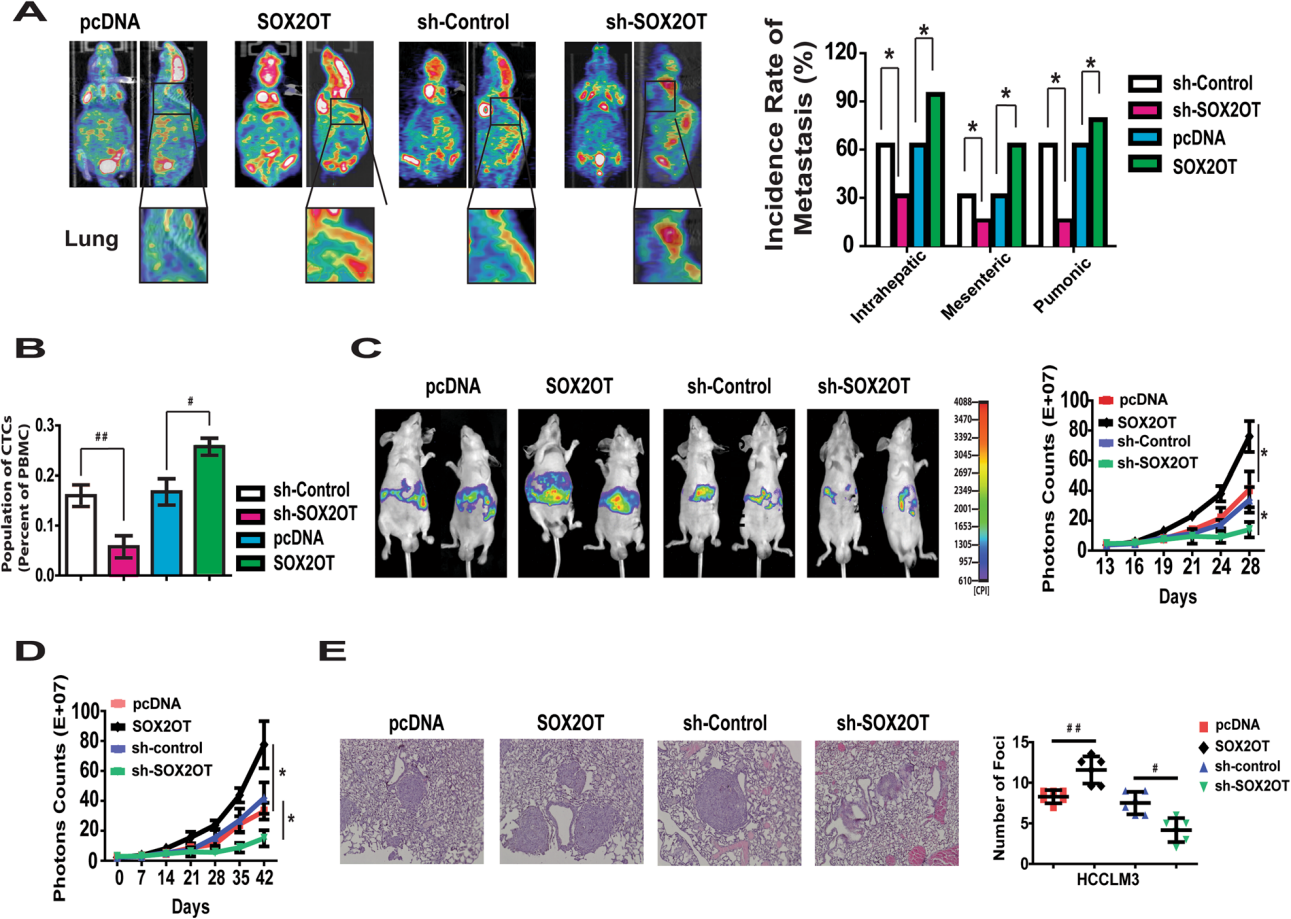
Effect of lncRNA-SOX2OT on in vivo tumor metastasis of HCC cells. **a** The metastatic potential of HCCLM3 orthotopic nude mice model with different lncRNA-SOX2OT level was evaluated by PET scan. SOX2OT overexpression enhanced intrahepatic, mesenteric and pulmonary metastasis. Conversely, the depletion of SOX2OT clocked metastasis. **b** Ectopic expression of lncRNA-SOX2OT significantly induced the number of CTCs (GFP labeled). The depletion of lncRNA-SOX2OT reduced the number of CTCs. **c** Overexpression of lncRNA-SOX2OT resulted in greater liver metastases burden, while the depletion of lncRNA-SOX2OT decreased the liver metastases burden in HCCLM3 cells. **d**, **e** lncRNA-SOX2OT overexpression increased the lung metastases burden, while the depletion of lncRNA-SOX2OT significantly reduced the lung metastases burden of HCCLM3 cells.

HCCLM3 cells stably expressing lncRNA-SOX2OT or control vector were injected subcutaneously into nude mice. The average tumor volume of HCCLM3 cells stably transfected with lncRNA-SOX2OT was much larger than tumor volume in the control group (Fig. S3A). To further evaluate the effect of lncRNA-SOX2OT on HCC metastasis, HCCLM3 tumor xenografts were isolated and implanted into the liver to establish orthotopic models. Then, PET scans were performed to evaluate the existence of metastasis. In this model system, lncRNA-SOX2OT overexpression effectively enhanced intrahepatic (66.7% vs. 33.3%), mesenteric (33.3% vs. 16.7%), and pulmonary metastases (66.7% vs. 16.7%) in HCCLM3 cells. Conversely, the depletion of lncRNA-SOX2OT blocked intrahepatic (66.7% vs. 100%), mesenteric (33.3% vs. 66.7%), and pulmonary metastases (66.7% vs. 83.3%) (Fig. 3a).

Because metastasis is a complex multistep process that involves the early step of tumor invasion and the late step of metastatic colonization in distant organs, we aimed to explore the influence of lncRNA-SOX2OT on the different stages of metastasis. First, orthotopic tumor models were established using HCCLM3 cells labeled with green fluorescent protein. The circulating tumor cells (CTCs) were examined 5 weeks later with flow cytometry. The results indicated that ectopic expression of lncRNA-SOX2OT significantly induced the number of CTCs (Fig. 3b). The depletion of lncRNA-SOX2OT reduced the number of CTCs.

To explore the influence of lncRNA-SOX2OT on liver colonization, we labeled HCCLM3 cells with luciferase and inoculated cells into nude mice via intrasplenic injection. Overexpression of lncRNA-SOX2OT resulted in greater liver metastases burden, while the depletion of lncRNA-SOX2OT decreased the liver metastases burden (Fig. 3c).

We further evaluated the role of lncRNA-SOX2OT in lung colonization by inoculating cells directly into the tail veins of nude mice. Consistently, lncRNA-SOX2OT overexpression increased the lung metastases burden, while the depletion of lncRNA-SOX2OT significantly reduced the lung metastases burden (Fig. 3d, e and Fig. S3B).

### Positive correlation between lncRNA-SOX2OT expression and glucose metabolism of HCC tumors and cells were observed

**Fig. 4:**
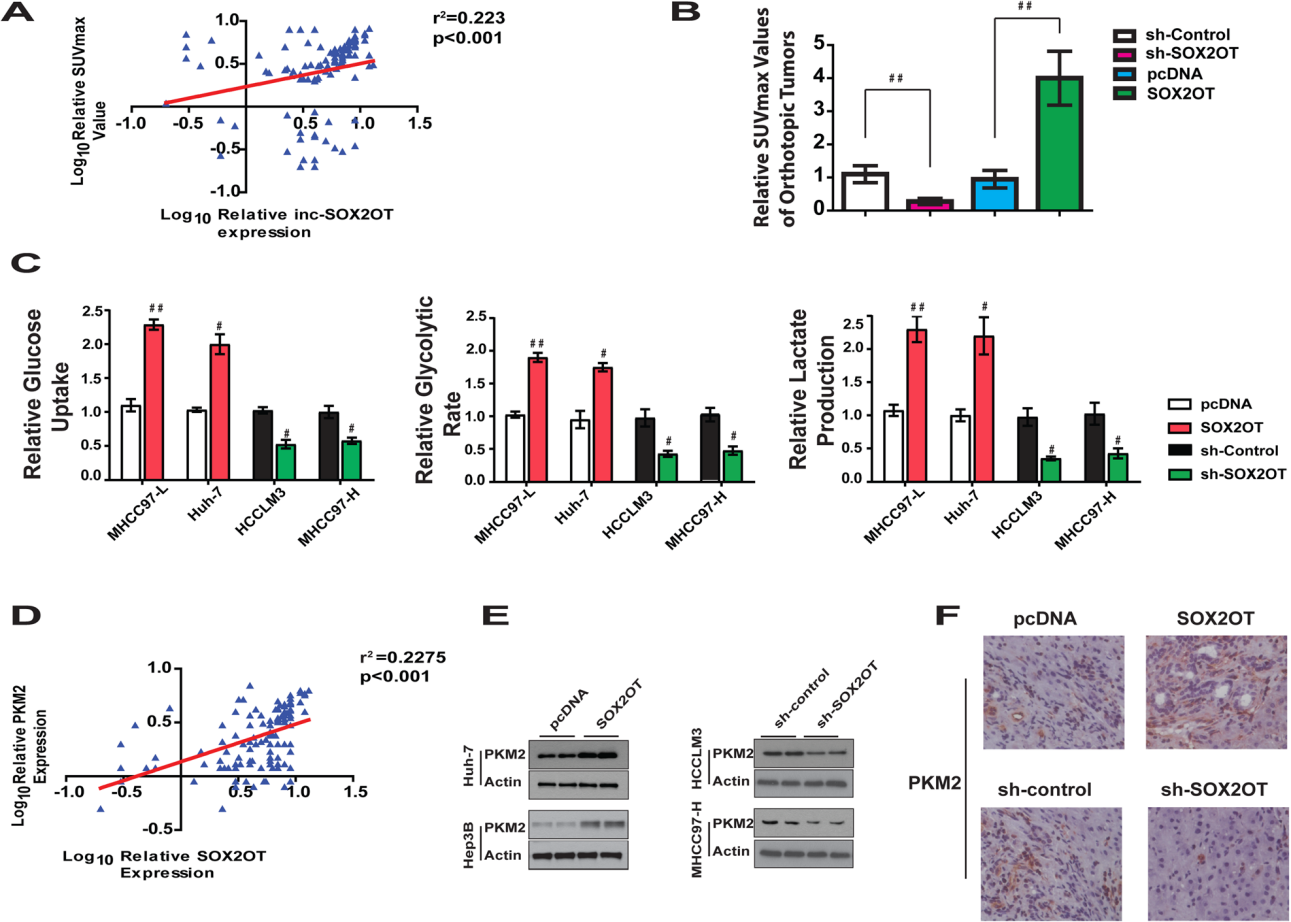
Positive correlation between lncRNA-SOX2OT expression and glucose metabolism of HCC tumors and cells. **a** Positive correlation was observed between lncRNA-SOX2OT expression and Suvmax value of HCC tumors. **b** The SUVmax value of orthotopic xenograft tumor constructed using HCCLM3 cells with lncRNA-SOX2OT overexpression was significantly higher than those tumors constructed using normal HCCLM3 cells or HCCLM3 cells with SOX2OT knockdown. **d** Overexpression of SOX2OT increased the level of glucose uptake, rate of glycolysis and lactate production in Huh-7 and MHCC97-L cells. Conversely, knockdown of SOX2OT resulted in opposite effects. **c** Positive correlation was observed between lncRNA-SOX2OT expression and PKM2 mRNA levels in HCC tumors. **d** lncRNA-SOX2OT promotes PKM2 protein level in Huh7 and Hep3B cells. Reciprocally, knockdown of lncRNA-SOX2OT inhibits PKM2 protein level in MHCC97-H and HCCLM3 cells. **e** lncRNA-SOX2OT promotes PKM2 expression in HCCLM3 orthotopic tumors.

To address the clinical relevance of lncRNA-SOX2OT expression and HCC glucose metabolism, we analyzed the correlation between lncRNA-SOX2OT mRNA level and PET SUVmax value in the 121 human patients with primary HCC. It was interesting to find that the PET SUVmax value was much higher in tumors that had a high level of lncRNA-SOX2OT expression. These data suggest a positive correlation between lncRNA-SOX2OT and carbohydrate metabolism in patients with HCC, which was further strengthened by Pearson analysis (Pearson *r* = 0.47, *p* < 0.00001; Fig. 4a). This result encouraged us to investigate whether lncRNA-SOX2OT enhances HCC metastasis through regulating the Warburg effect, which is characterized by higher glucose uptake, a higher rate of glycolysis, and higher lactate production in cancer cells than normal cells^23,24^.

This notion was first proofed by evaluating the SUVmax value of orthotopic xenograft tumors constructed using HCCLM3 cells with different expression levels of lncRNA-SOX2OT. The results indicated a positive correlation between lncRNA-SOX2OT and the SUVmax value of orthotopic xenograft tumors (Figs. 3a and 4b). The tumors with lncRNA-SOX2OT overexpression tended to demonstrate higher SUVmax value, while tumors with lncRNA-SOX2OT knockdown showed significantly lower SUVmax value.

Then, in vitro analysis was performed to further evaluate the correlation between lncRNA-SOX2OT expression and level of glucose metabolism. As shown in Fig. 4c, ectopic expression of lncRNA-SOX2OT in MHCC97-L and Huh-7 cells significantly increased glucose uptake, the rate of glycolysis, and lactate production. Opposite results could be observed after knockdown of endogenous lncRNA-SOX2OT in HCCLM3 and MHCC97-H cells.

The role of lncRNA-SOX2OT in mitochondrial respiration was also investigated. We found that lncRNA-SOX2OT overexpression or knockdown did not change oxygen consumption or ATP level (Fig. S4A, B). This evidence suggests that the alteration of lncRNA-SOX2OT expression in HCC cells is mainly responsible for the regulation of glycolysis, rather than mitochondrial respiration.

### PKM2 contributes to the function of lncRNA-SOX2OT in regulating glucose metabolism and the Warburg effect

To explore the target that contributes to the Warburg effect of HCC induced by lncRNA-SOX2OT, the mRNA levels of a series of enzymes crucial to glycolysis were evaluated. No clear correlation was observed with HK1, PFK, HK2, LDHA, and pyruvate dehydrogenase (Fig. S4C). However, a significant positive correlation between lncRNA-SOX2OT and PKM2 expression was observed (Fig. 4d). When the protein levels were evaluated, a positive correlation was found between lncRNA-SOX2OT expression and PKM2 protein level (Fig. S4D), indicating that lncRNA-SOX2OT might regulate HCC glucose metabolism by modifying the level of PKM2. To confirm this hypothesis, we measured the protein expression levels of PKM2 by western blot analysis in Huh-7 and Hep3B cells with lncRNA-SOX2OT overexpressed, or in HCCLM3 and MHCC97-H cells with lncRNA-SOX2OT under-expressed. The results in Fig. 4e shows that the expression of PKM2 was significantly higher in cells stably transfected with lncRNA-SOX2OT than with pcDNA, and was significantly reduced in the lncRNA-SOX2OT knockdown group compared with the control group. These findings suggest that lncRNA-SOX2OT can promote PKM2 protein level. Next, immunofluorescence staining was performed to further investigate the effect of lncRNA-SOX2OT on PKM2 expression in HCCLM3 cells stably transfected with lncRNA-SOX2OT or pcDNA. Consistent with the results of western blot, the data showed that lncRNA-SOX2OT could promote PKM2 protein level (Fig. S4E).

To examine the promoting effect of lncRNA-SOX2OT on PKM2 in vivo, liver tumor samples from orthotopic liver cancer xenograft models constructed with HCCLM3 cells with different levels of lncRNA-SOX2OT were collected for immunohistochemical assay. The results indicated that the PKM2 protein levels in the lncRNA-SOX2OT overexpression group were significantly higher than that of the control group (Fig. 4f). lncRNA-SOX2OT knockdown decreased the level of PKM2. These results demonstrate that PKM2 might be a target of lncRNA-SOX2OT in regulating the Warburg effect.

### lncRNA-SOX2OT promotes the expression of PKM2, glucose metabolism, and metastasis in HCC cells, and is physically associated with miR-122-5p

**Fig. 5:**
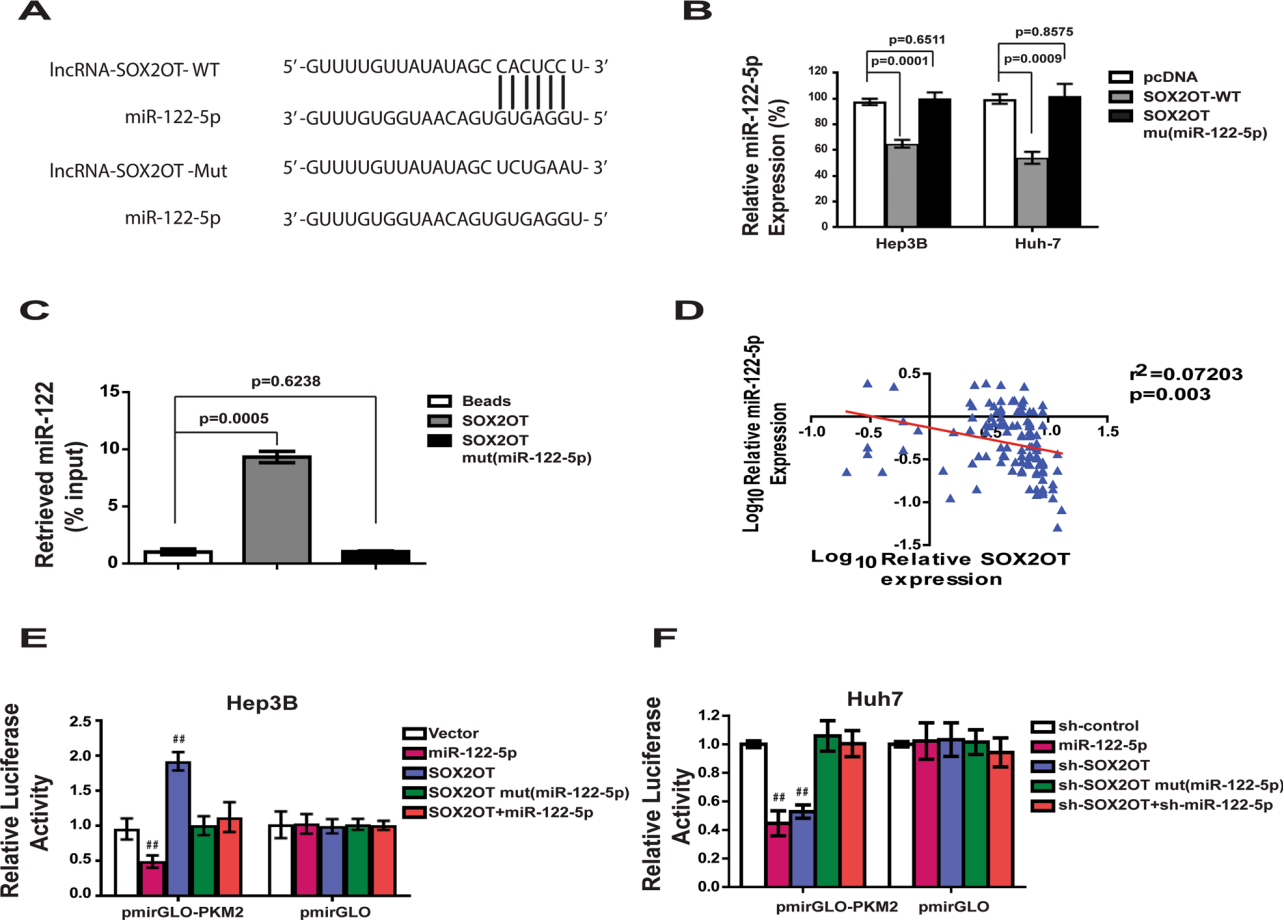
miR-122-5p is a direct target of lncRNA-SOX2OT. **a** Schematic outlining the predicted binding sites of miR-122-5p on lncRNA-SOX2OT WT. Construct mutant lncRNA-SOX2OT: lncRNA-SOX2OTMut. **b** Ectopically expressed lncRNA-SOX2OT WT, but not the mutant, reduced the levels of miR-122-5p at endogenous levels in Hep3B and Huh-7 cells. **c** Huh7 cell lysates were incubated with biotin-labeled lncRNA-SOX2OT; after pull-down, miR-122-5p was extracted and assessed by qRT-PCR. **d** Reverse correlation of lncRNA-SOX2OT and miR-122-5p expression was observed. **e**, **f** Luciferase activity assay. Overexpression of lncRNA-SOX2OT WT, but not the mutant, increased the luciferase activity of pmirGLO-PKM2. Ectopic expression of miR-122-5p abolished the upregulation. Consistent results can be obtained from SOX2OT knockdown assay.

Recently, many RNA transcripts have been reported to function as competing endogenous RNAs (ceRNA) by competitively binding common microRNAs. To identify microRNAs responsible for the regulation of PKM2 by lncRNA-SOX2OT, the LncBase prediction algorithm (LncBase Predicted v.2) was used to predict the lncRNA-miRNA binding. Interestingly, the miR-122-5p microRNA, which has lncRNA-SOX2OT binding sites (Fig. 5a), also has PKM2 targeting site (Fig. S5A). To investigate the potential regulating effect of miR-122-5p, lncRNA-SOX2OT-mut(miR122-5p) with mutations in the miR-122-5p targeting site was constructed. Our results indicated that ectopically expressed lncRNA-SOX2OT WT, but not the mutant, reduced the levels of miR-122-5p at endogenous levels in Hep3B and Huh-7 cells (Fig. 5b). Furthermore, the specific association between miR-122-5p and lncRNA-SOX2OT was validated in Huh-7 cells by affinity pull-down of endogenous miR-122-5p using in vitro transcribed biotin-labeled lncRNA-SOX2OT. After pull-down by lncRNA-SOX2OT WT, the level of miR-122-5p was much higher than that of the control group (Fig. 5c). lncRNA-SOX2OT-mut(miR122) did not pull down miR-122-5p.

To confirm the correlation between lncRNA-SOX2OT and miR122, the expression of miR-122-5p in 105 HCC patients was evaluated using real-time PCR. The results of Pearson analysis indicated a significant reverse correlation of lncRNA-SOX2OT and miR-122-5p expression (Pearson *r* = −0.456, *p* < 0.00001; Fig. 5d). To further confirm whether miR-122-5p mediated the function of lncRNA-SOX2OT promoting PKM2 expression, a dual-luciferase reporter system was used. As shown in Fig. 5e, overexpression of lncRNA-SOX2OT WT, but not the mutant, increased the luciferase activity of pmirGLO-PKM2. Ectopic expression of miR-122-5p abolished the upregulation. Reciprocally, the depletion of lncRNA-SOX2OT decreased the luciferase activity of pmirGLO-PKM2, which was reduced by inhibition of miR-122-5p (Fig. 5f). Taken together, these findings show that miR-122-5p is a potential target of lncRNA-SOX2OT in regulating PKM2 level.

### miR-122-5p mediates the role of lncRNA-SOX2OT in regulating PKM2, glucose metabolism, and HCC cell metastasis

**Fig. 6:**
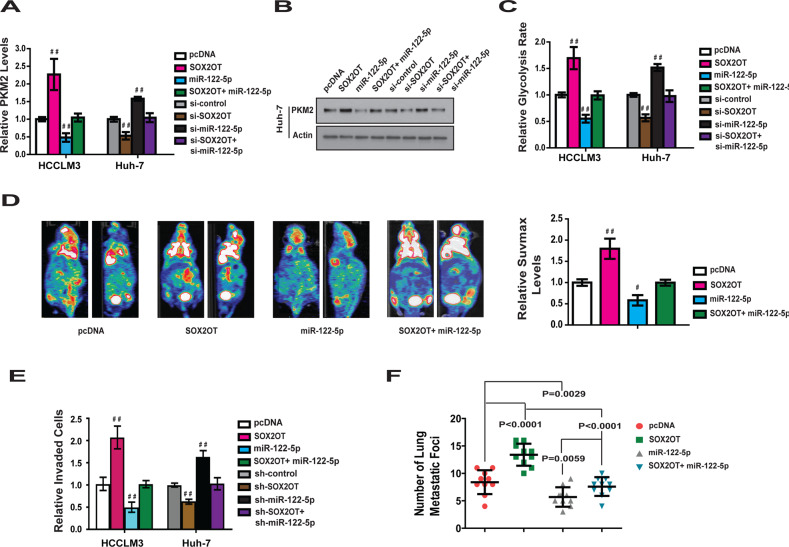
miR-122-5p mediates the role of lncRNA-SOX2OT in regulating PKM2, glucose metabolism and HCC cell metastasis. **a** Overexpression of miR-122-5p blocks the promotion of PKM2 mRNA by SOX2OT. On the contrary, knockdown of mR-122 attenuated the inhibition of PKM2 by shRNA- SOX2OT. **b** The change of PKM2 protein level in HCCLM3 cells after overexpression and knockdown the expression of lncRNA-SOX2OT or miR-122-5p. **c** The rate of glycolysis in HCCLM3 cells was enhanced by SOX2OT overexpression and this enhancement could be blocked by overexpression of miR-122-5p. On the contrary, knockdown of mR-122 attenuated the inhibition of glycolysis rate by shRNA- SOX2OT. **d** The value of SUV max in lncRNA-SOX2OT overexpression orthotopic nude mice liver tumor was much higher than control group. Reciprocally, the increase of SUV max value was blocked by ectopic expression of miR-122-5p. **e** The promoting effect of lncRNA-SOX2OT on HCC cell invasion was antagonized by miR-122-5p overexpression. One the contrary, knockdown of miR-122-5p attenuated the inhibition effect of shRNA-lncRNA-SOX2OT. **f** The total number of lung metastatic lesions in the lncRNA-SOX2OT over-expression lung metastasis model was much more than that of control group. However, this increase can be blocked by overexpression of miR-122-5p.

To confirm that miR-122-5p contributed to the function of lncRNA-SOX2OT in regulating metabolism and metastasis of HCC, the expression of miR-122-5p was knocked down in Huh-7 cells and overexpressed in HCCLM3 cells (Fig. S6A). We found that overexpression of miR-122-5p blocked the promotion of PKM2 by lncRNA-SOX2OT (Fig. 6a). Knockdown of mR-122 attenuated the inhibition of PKM2 by shRNA-lncRNA-SOX2OT (Fig. 6a). Similar changes were observed in PKM2 protein level (Fig. 6b).

We then found that miR-122-5p contributes to the role of lncRNA-SOX2OT in regulating glucose metabolism. As shown in Fig. 6c and Fig. S6B, ectopic expression of miR-122-5p blocked the effect of lncRNA-SOX2OT in promoting glucose uptake, glycolysis, and lactate production. Knockdown of endogenous miR-122-5p attenuated the effect of shRNA-lnRNA-SOX2OT in inhibiting glucose metabolism.

These results were further confirmed in nude mice orthotopic liver cancer xenograft models constructed using HCCLM3 control cells, HCCLM3 lncRNA-SOX2OT-overexpressing cells, HCCLM3 miR-122-5p-overexpressing cells, and HCCLM3 simultaneous lncRNA-SOX2OT- and miR-122-5p-overexpressing cells. PET-CT scans were performed to evaluate the level of glucose metabolism in the tumors. The results demonstrated that the value of SUVmax in the lncRNA-SOX2OT overexpression group was much higher than that in the control group (Fig. 6d). The increase in SUVmax value was blocked by ectopic expression of miR-122-5p. Taken together, these results suggest that miR-122-5p is an important mediator for lnRNA-SOX2OT’s role in regulating glucose metabolism.

The effect of lncRNA-SOX2OT on HCC cell metastasis was also evaluated. The results (Figs. 6e, S6C) indicated that the promoting effect of lncRNA-SOX2OT on HCC cell invasion was antagonized by miR-122-5p overexpression. Knockdown of miR-122-5p attenuated the inhibiting effect of shRNA-lncRNA-SOX2OT on invasion of HCCLM3 and Huh-7 cells. This effect was further confirmed in a nude mouse model of lung metastasis. Consistent with the in vitro results, the total number of lung metastatic lesions in the lncRNA-SOX2OT overexpression group was greater than that of control group. However, this increase was blocked by overexpression of miR-122-5p (Fig. 6f). These results indicated that miR-122-5p contributes to the function of lncRNA-SOX2OT in regulating metastasis of HCC in vitro and in vivo.

### lncRNA-SOX2OT promotes HCC invasion through regulating EMT

EMT has been shown to be of critical importance in the early events of tumor cell metastatic. To investigate whether lncRNA-SOX2OT regulates HCC metastasis through modulating EMT, the expression of epithelial markers E-cadherin and ZO-1 and mesenchymal markers N-cadherin and vimentin was examined. Our results indicated that overexpression of lncRNA-SOX2OT, but not the mutant, reduced E-cadherin and ZO-1 and increased N-cadherin and vimentin (Fig. S7A, B). Consistently, ectopic expression of miR-122-5p abolished the effect. Reciprocally, the depletion of lncRNA-SOX2OT induced an epithelial phenotype (Fig. S7C, D).

Immunofluorescence staining revealed that overexpression of lncRNA-SOX2OT blocked increased E-cadherin and ZO-1 expression in the cell membrane, and induced N-cadherin and vimentin. Conversely, the depletion of lncRNA-SOX2OT induced an epithelial phenotype, upregulated E-cadherin and ZO-1, and downregulated N-cadherin and vimentin (Fig. S7E).

We next investigated the occurrence of EMT in vivo. As analyzed by immunohistochemistry, lncRNA-SOX2OT overexpression exhibited the inhibition of the typical EMT phenotype, including focal increment of the epithelial marker E-cadherin and concurrent loss of vimentin and N-cadherin (Fig. S7F). Similarly, ectopic expression of miR-122-5p abolished the effect. Together, these findings show that lncRNA-SOX2OT promotes HCC invasion through regulating EMT.

## Discussion

Recent studies have shown that metabolic changes are a hallmark of tumor cells and a key contributor to tumor development^4,26^. Emerging evidence has indicated that the Warburg effect contributes greatly to tumorigenesis and could be targeted for tumor therapy^27,28^. In HCC, increased glycolysis also plays an essential role in supporting tumor development. Numerous HCC tumors appeared to have increased expression of glycolytic genes, such as PKM2, hexokinase, LDHA, and GLUT-1; modulation of these genes could affect HCC cell growth^29,30^. A recent report showed that patients with HCC whose tumors had higher glucose uptake had shorter survival times^31^. However, the impact of the Warburg effect on HCC metastasis remains largely unknown.

An important idea of metabolic therapy is to identify the key biochemical nodes in the dysregulated cancer metabolism. In recent years, lncRNAs, have been found to regulate the biological processes of tumors through a variety of mechanisms. However, there is little understanding of crucial lncRNAs functioning downstream of the metabolic pathway.

In the present study, the SUVmax value was much higher in HCC tumors with relatively higher levels of lncRNA-SOX2OT expression. Furthermore, ectopic expression of lncRNA-SOX2OT in Hep3B and Huh7 cells significantly promoted glucose uptake, the rate of glycolysis, and lactate production. Knockdown of endogenous lncRNA-SOX2OT in HCCLM3 and MHCC97-H cells produced the opposite effect. These data suggest a positive correlation between lncRNA-SOX2OT and glucose metabolism in patients with HCC. However, neither exogenous lncRNA-SOX2OT expression nor lncRNA-SOX2OT knockdown changed the oxygen consumption and ATP level in HCC cells. This finding suggests that the alteration of lncRNA-SOX2OT expression in HCC cells is mainly responsible for the regulation of glycolysis, rather than mitochondrial respiration.

We further found that lncRNA-SOX2OT promotes HCC glucose metabolism by targeting PKM2, one of the most important enzymes in the glycolytic pathway. PKM2 plays a key step early in glycolysis, phosphorylating glucose to produce glucose-6-phosphate. A substantial amount of data supports PKM2 as a molecular target for the diagnosis and treatment of cancer^32^. Our data suggest that lncRNA-SOX2OT upregulation in HCC cells may result in enhanced PKM2 levels in the HCC microenvironment, which subsequently activate the glycolytic pathway and thereby promote tumorigenesis.

Pro-metastatic activity is another function of lncRNA-SOX2OT that we identified in the present study. Recently, one group identified a lncRNA-Dreh which is downregulated in hepatitis B virus (HBV)-HCC can inhibit HCC growth and metastasis in vitro and in vivo, playing a role similar to that of a tumor suppressor^22^. Herein, we reported that lncRNA-SOX2OT promotes HCC metastasis, based on observations from human specimens and in vitro and in vivo models. We presented evidence that the induction of lncRNA-SOX2OT expression markedly increased the intrahepatic and pulmonary metastasis of orthotopic xenograft HCC tumors, and that the downregulation of lncRNA-SOX2OT in human HCC tissues was associated with attenuated metastasis.

These results prompted consideration that the induction of glucose metabolism may have contributed to the promoting effect of lncRNA-SOX2OT against HCC metastasis. To further confirm this notion, the expression of PKM2, which mediated the suppression of glucose metabolism of lncRNA-SOX2OT, was knocked down. Our studies revealed that PKM2 is also the main mediator of lncRNA-SOX2OT in regulating HCC metastasis. The upregulation of PKM2 in HCC may contribute to tumor metastasis caused by lncRNA-SOX2OT, at least in part, through the activation of glucose metabolism.

To further illustrate the regulation of PKM2 by lncRNA-SOX2OT, RNA pull-down and real-time PCR analysis were performed. MiR-122-5p was found to bind with lncRNA-SOX2OT directly and regulate the level of PKM2. This finding was further verified in our in vitro system, the xenograft metastasis model, the xenograft metabolism model, and clinical HCC tissues. MiR-122-5p contributes to the induction of PKM2 by lncRNA-SOX2OT and further inhibits metabolism-mediated HCC metastasis. Our finding is consistent with the previous notion that the increased glycolysis results in acute and chronic acidification of the local environment through the conversion of pyruvate to lactic acid^33^. This micro-environmental acidosis leads to invasion and metastasis through inhibition of gap-junction conductance, and, probably, activation of metalloproteinases promoting the degradation of the extracellular matrix and participating in EMT^34^. Interestingly, our results further demonstrated that lncRNA-SOX2OT enhanced the EMT phenotype of HCC cells by regulating the expression of EMT regulators and markers, such as E-cadherin, ZO-1, N-cadherin, and vimentin.

In conclusion, lncRNA-SOX2OT is upregulated in HCC. lncRNA-SOX2OT promotes HCC metastasis by upregulating PKM2, which increases the glycolytic pathway in HCC cells and thereby enhances EMT. Therefore, lncRNA-SOX2OT could function as a metastasis enhancer in HCC. These findings are an important supplement to the underlying mechanism of HCC metastasis and provide a new lncRNA target for the treatment of HCC metastasis.

## Materials and methods

### Patients

Frozen HCC tissues, normal liver tissues, and PVTT tissues were randomly obtained with informed consent from patients who underwent radical resections in the First Affiliated Hospital of Harbin Medical University. After selection, 121 HCC patients were enrolled in this study and informed consent was obtained from all patients. Ethical consent was granted from the Ethics Committee of the First Affiliated Hospital of Harbin Medical University, Harbin, China. Patients enrolled in our study received neither radiation therapy nor chemotherapy prior to surgery.

### Western blot analysis

Standard western blot assays were used to analyze protein expression, as described^33^. Antibodies against E-cadherin, ZO-1, N-cadherin, vimentin, and β-actin were obtained from Abcam. Antibodies against HK1, HK2, PFK, PKM2, and LDHA were purchased from Cell Signaling Technology. Information of antibodies used can be found in Supplementary Table 2.

### Statistical analysis

Statistical analysis was performed with the GraphPad Prism software package (v. 4.02; San Diego, CA, USA) or SPSS 16.0 software (Chicago, IL, USA). Results are presented as mean values ± SD of at least three different experiments performed in triplicate. The survival curves were plotted using the Kaplan–Meier method. Student’s *t* test or one-way ANOVA was applied to determine the significance between groups, and *p* < 0.05 was considered statistically significant (^*^p < 0.05, ^#^*p* < 0.01, ^##^*p* < 0.001).

## Supplementary information

Supplementary Table 1

Supplementary materials, methods and figure legends

Supplementary Table 2

Supplementary Figure 1

Supplementary Figure 2

Supplementary Figure 3

Supplementary Figure 4

Supplementary Figure 5

Supplementary Figure 6

Supplementary Figure 7
