# Supplementary material for: lncRNA-SOX2OT promotes hepatocellular carcinoma invasion and metastasis through miR-122-5p-mediated activation of PKM2
Source: Oncogenesis. 2020 May 28;9(5):54. doi: 10.1038/s41389-020-0242-z (PMC7256049; doi:10.1038/s41389-020-0242-z)
Supplement: Supplementary file 1 — Supplementary Table 1 [file 41389_2020_242_MOESM1_ESM.doc]

Supplementary Table 1. Characteristics of 105 HCC patients with different metastatic potential.

| Variabls | Metastatic potential | |  |
| --- | --- | --- | --- |
|  | High (n=51) | Low (n=54) |  |
|  | n(%) | n(%) | *P* value |
| Age (years) |  |  |  |
| ≤50 | 26(50.98%) | 25(46.30%) | 0.776 |
| ＞50 | 25(49.01%) | 29(53.70%) |  |
| Gender |  |  |  |
| Male | 28(54.90%) | 30(55.56%) | 0.897 |
| Female | 23(45.10%) | 24(44.44%) |  |
| HBsAg |  |  |  |
| Negative | 11(21.57%) | 13(24.07%) | 0.942 |
| Positive | 40(78.43%) | 41(75.93%) |  |
| Anti-HCV |  |  |  |
| Negative | 49(96.08%) | 52(96.30%) | 0.651 |
| Positive | 2(3.92%) | 2(3.70%) |  |
| Liver cirrhosis |  |  |  |
| No | 6(11.76%) | 5(9.26%) | 0.920 |
| Yes | 45(88.24%) | 49(90.74%) |  |
| Serum AFP, ng/ml |  |  |  |
| ≤20 | 14(27.45%) | 13(24.07%) | 0.863 |
| ＞20 | 37(72.55%) | 41(75.93%) |  |
| Tumor diameter (cm) |  |  |  |
| ≤5 | 35(68.63%) | 37(68.52%) | 0.843 |
| ＞5 | 16(31.37%) | 17 (31.48%) |  |
| TNM stage |  |  |  |
| I | 19(37.25%) | 21(38.89%) | 0.024 |
| II -Ⅳ | 32(62.75%) | 33(61.11%) |  |
|  |  |  |  |

Abbreviations:AFP, alpha-fetoptotein; HBsAg, hepatitis B surface antigen; HCV, hepatitis C virus.
