# Supplementary material for: lncRNA-SOX2OT promotes hepatocellular carcinoma invasion and metastasis through miR-122-5p-mediated activation of PKM2
Source: Oncogenesis. 2020 May 28;9(5):54. doi: 10.1038/s41389-020-0242-z (PMC7256049; doi:10.1038/s41389-020-0242-z)
Supplement: Supplementary file 2 — Supplementary materials, methods and figure legends [file 41389_2020_242_MOESM2_ESM.docx]

**Supplementary Materials and Methods**

**Cell lines and culture conditions**

The WRL68 immortalized liver cell line was purchased from ACCEGEN (NJ, USA). Huh-7 and Hep3B human HCC cells were purchased from the American Type Culture Collection (Manassas, VA, USA). All cell lines have been authenticated by STR profiling. In order to compare lncRNA-SOX2OT levels in HCC cell lines with different metastatic potential, we selected the human liver cancer cell lines MHCC97L, MHCC97H and HCCLM3, which respectively display stepwise, progressively increasing metastatic potential (Institute of Biochemistry and Cell Biology, Chinese Academy of Science, China.) All cells were cultured at 37 °C in an atmosphere containing 5% CO_2_ and in Dulbecco’s modified Eagle’s medium (Invitrogen, Carlsbad, CA) supplemented with 10% fetal bovine serum.

**Microarray analysis**

As described previously^[21]^, the total RNA extracted from patient HCC tissue and normal liver tissue was amplified and transcribed into fluorescent cRNA using the Quick Amp Labeling kit (Agilent Technologies, Palo Alto, CA). The labeled cRNA was then hybridized onto the Human lncRNA Array v2.0 (8 x 60K, ArrayStar, Rockville, MD). After the washing steps, the arrays were scanned by the Agilent Scanner G2505B. Agilent Feature Extraction software (version 10.7.3.1) was used to analyze acquired array images. Quantile normalization and subsequent data processing were performed using the GeneSpring GX v11.5.1 software package (Agilent Technologies). The differentially expressed lncRNAs with statistical significance were identified using volcano plot filtering. The threshold that we used to screen upregulated or downregulated lncRNAs is fold change ≥ 1.5 and a *p*-value ≤ 0.05. Gene expression profiles were determined using Phalanx human OneArray microarrays (HOA 6.1), following the manufacturer's instructions.

**RNA pull-down**

As described previously^[21]^, lnRNA-SOX2OT and lncRNA-SOX2OT-mut(miR-122-5p) were *in vitro* transcribed respectively from vector pSPT19-SOX2OT and pSPT19-SOX2OT-mut(miR-122-5p), and were biotin-labeled with the Biotin RNA Labeling Mix (Roche) and T7 RNA polymerase (Roche). This was followed by treatment with RNase-free DNase I (Roche) and purification with a RNeasy Mini Kit (Qiagen, Valencia, CA). One milligram of whole-cell lysates from Huh7 cells was incubated with 3ug of purified biotinylated transcripts for 1h at 25 °C; complexes were isolated with streptavidin agarose beads (Invitrogen). The RNA present in the pull-down material was detected by qRT-PCR analysis.

**RNA extraction and real-time PCR**

As described previously^[23]^, total RNA was isolated using Trizol reagent (Invitrogen). First-strand cDNA was generated using the M-MLV Reverse Transcriptase (Invitrogen) and either gene-specific primers or random primers. Real-time PCR was performed in the StepOne™ Real-Time PCR System (Applied Biosystems, Foster City, USA) using SYBR® Green (Takara, Dalian, China). The PCR primers for lncRNA SOX2OT, miR-122-5p, PKM2, or GAPDH were as follows: lncRNA SOX2OT sense, 5’-GCTCGTGGCTTAGGAGATTG-3’ and reverse, 5’-CTGGCAAAGCATGAGGAACT-3’; miR-122-5p sense: 5’-CGGTAGCAGCACATAATCG-3’ and reverse, 5’-GTGCAGGGTCCGAGGA-3’; PKM2 sense: 5’-GCACTCTGACTGA-TTCTCTGG-3’ and reverse, 5’-GCACTCTGACTGATTCTCTGG-3’; GAPDH sense, 5’-GTCAACGGATTTGGTCTGTATT-3’ and reverse, 5’-AGTCTTCTGGGTG- GCAGTGAT-3’. Real-time PCR was performed as above, using TaqMan microRNA assays according to the manufacturer’s instructions (Applied Biosystems). The relative expression of RNAs was calculated using the comparative Ct method.

**Measurement of Glucose Uptake**

As described previously^[23]^, glucose uptake was measured by determining the uptake of 2-[3H] deoxyglucose by cells, as described previously^[24]^. In brief, cells (1×10^5^) were seeded onto 12-well plates and cultured for 12 h. Cells were then washed twice with PBS and preincubated in glucose-free medium for 30 min. Then, 2-[3H] deoxyglucose was added to the cells and cells were incubated for 30 min at 37 °C. Uptake of 2-[3H] deoxyglucose was terminated by a rapid removal of medium, followed by washing with PBS three times. The cells were then lysed in 300 μL of 1% SDS, and the radioactivity of collected cell lysates was determined in a Beckman LS 6000 SC liquid scintillation counter (Beckman Coulter). Glucose uptake was normalized to the protein concentrations of the cell lysates.

**Measurement of the Rate of Glycolysis**

The rate of glycolysis was measured by monitoring the conversion of 5-[3H] glucose to 3H_2_O as described^[23]^. In brief, cells (1×10^6^) were collected and washed in PBS once and then resuspended in 1 mL of Krebs buffer without glucose for 30 min at 37 °C. Cells were then collected and resuspended in 0.5 mL of Krebs buffer containing 10 mM glucose and 5 μCi of 5-[3H]glucose for 1 h at 37 °C. Triplicate 100-μL aliquots were transferred to uncapped PCR tubes containing 100μL of 0.2 NHCl, and a tube was transferred to a scintillation vial containing 0.5 mL of H_2_O. The scintillation vials were sealed and left for 48 h to allow diffusion to occur. The amounts of diffused and undiffused 3H were determined in a Beckman LS 6000 SC liquid scintillation counter (Beckman Coulter). The rate of glycolysis was calculated as described.

**Measurement of Lactate Production**

As described previously ^[23]^, lactate levels in the culture medium of cells were determined by using a Lactate Assay Kit (Biovision). Cells (1×10^5^) were seeded onto 12-well plates for 12h and were then replenished with fresh phenol red-free medium (Sigma). Cells were then incubated for 16–24 h, and the culture medium was collected for measurement of lactate concentrations. Lactate levels were determined by using a Lactate Assay Kit (Biovision), and normalized with cell number.

**Transfection**

Cell transfection assay was performed as described previously^[21]^. Briefly, short hairpin RNA (shRNA) against SOX2OT, HK1, HK2，PKM2, LDHA or PFK genes, as well as their non-targeting sequences were constructed in specific shRNA vector (Gene Pharama, Shanghai, China). MiR-122 agomir, miR-122 antagomir and their respective negative control were synthesized (GenePharama, Shanghai, China).

**Luciferase reporter assay**

Luciferase reporter assay was performed as described previously^[21]^. Briefly, the sequence of SOX2OT was amplified by PCR and cloned into pmirGLO Dual-luciferase miRNA Target Expression Vectors, along with its mutant sequence of mir-122-5p binding sites (GenePharma, Shanghai, China). Hep3B and Huh7 cells were seeded in a 96-well plate (Corning) and co-transfected with wild-type pmirGLO-SOX2OT or SOX2OT mutant reporter plasmid and mir-122-5p mimic or miR-122-5p-NC, respectively. The relative luciferase activity was normalized to Renilla luciferase activity 48 h after transfection.

**Immunofluorescence Analysis**

Immunofluorescence analysis was performed as described previously ^[23]^. Briefly, different stable Huh7 cell clones were cultured and fixed on 12×12 mm glass slides. Cells were first incubated with antibodies specific for E-cadherin (Abcam), ZO-1 (Invitrogen), N-cadherin (BD Transduction Laboratories), vimentin (Cell Signaling Technology), or β-tubulin (Cell Signaling Technology) and were then incubated with goat anti-rat IgG (Alexa Fluor 594, Invitrogen), goat anti-mouse IgG (Alexa Fluor 488, Invitrogen), or goat anti-rabbit IgG (Alexa Fluor 594, Invitrogen). The slides were mounted by adding DAPI-Fluoromount-G (Southern Biotech, SBA, Birmingham, AL), and were examined with a Zeiss axiophot photomicroscope (Carl Zeiss, Oberkochen, Germany).

**Invasion assays**

Cell migration assays were performed in the BD Falcon 24-multiwell insert system (BD Biosciences, San Jose, CA, USA), as previously described ^[23]^. For the matrigel invasion assay, filters were precoated with 30 μl Matrigel (BD Biosciences, USA) for 3 h. Culture medium containing 10% FBS was added to lower chambers, and aliquots of 1-3 × 10^4^ cells in 300 μl of FBS-free medium were seeded into upper chambers. Then, non-migrated or non-invaded cells were removed from the upper surface of the filter. Cells on the lower surface of the membrane were fixed with ice-cold methanol and stained with crystal violet. Cell numbers were counted under an optical microscope (×100). Each experiment was repeated at least three times.

**Animal studies**

All mice were obtained from the laboratory animal center of the Chinese academy of sciences, Shanghai. The experimental protocol was reviewed and approved by the Committee on the Use of Live Animals in Teaching and Research of the Harbin Medical University, Harbin, China (SYSK 2010-012). The procedures were performed as described previously ^[23]^ and no randomization or blinding was used. Briefly, for subcutaneous experiment, male BALB/c (5-6 weeks old) mice (n=6/group) were inoculated subcutaneously in the flank with 2×10^6^ HCCLM3 cells suspended in PBS. Tumor size was measured twice weekly using a digital caliper. The tumor volume was calculated using the formula: length × (width) ^2^ × 0.52 and subsequently transformed into relative values (V; V = Vt/V0, where V0 is the tumor volume at initiation of treatment and Vt is the tumor volume at any given day during entire treatment period). After subcutaneous tumors were first constructed, orthotopic liver tumor model was introduced. Pieces of the subcutaneous tumors were cut into 1-mm^3^ sections. An upper-abdominal incision was made while the mouse was anesthetized. The left lobe of the liver was exposed and part of the liver surface was mechanically injured with scissors. A tumor piece was fixed in the liver tissue, the liver was returned to the peritoneal cavity, and the abdominal wall was closed. The mice were kept in pathogen-free conditions. An intrasplenic injection model (n=6/group) was used for liver colonization assays. A tail vein injection model (n=6/group) was used for lung colonization assays. The metastases were monitored using the IVIS@ Lumina II system (Caliper Life Sciences, Hopkinton, MA) 10 min after intraperitoneal injection with 4.0 mg of luciferin (Gold Biotech) in 50 μl of saline. Survival was recorded daily. After 10 weeks, the mice were sacrificed, and their lungs were dissected and prepared for standard histological examination. Lung metastatic progression or nodules were monitored and quantified either using the noninvasive bioluminescence system or by counting under the dissecting microscope.

**Flow cytometry**

As described previously^[23]^, whole blood samples were collected from mice 5 weeks after orthotopic xenografting using different HCCLM3 cell clones. Peripheral blood mononuclear cells (PBMCs), including circulating tumor cells (CTCs) were enriched from the blood. PBMCs from mice without tumor were used as negative controls to exclude the influence of autofluorescence and to select the appropriate gate in flow cytometry. GFP-positive CTCs were then counted by using the selected gate. Flow cytometric analysis was performed with a FACS Calibur flow cytometer (BD Biosciences, San Jose, CA).

**Immunohistochemistry analysis**

Immunohistochemistry was performed as described previously^[23]^, using E-cadherin, ZO-1, N-cadherin, and vimentin antibodies. In brief, tissue sections were deparaffinized in xylene and rehydrated with ethanol. Tissue sections were then preincubated with 10% normal goat serum in PBS (pH 7.5), followed with incubation with primary antibody overnight at 4 °C. Tissue sections were then stained with biotinylated secondary antibody (Vector lab) for 1 h at room temperature, followed by the Vectastain Elite ABC reagent (Vector lab) for 30 min. The peroxidase reaction was developed with diaminobenzidine (DAB kit; Vector lab), and the slides were counterstained with hematoxylin (Sigma).

***Supplementary Figure Legends***

***Figure S1 related to Figure 1. The level of metabolism is positively correlate with metastatic potential of HCC***

1. Representative PET-CT scan results of liver and lung in HCC patients having different metastatic outcomes.
2. Relative HK2, LDHA, HK1 and PFK mRNA levels of 5 HCC cell lines compared with normal liver cell line WRL68. Significant elevation of HK2 and LDHA expression could be observed in HCC cell lines. (## p<0.001)
3. Overexpression of HK2, PKM2, and LDHA markedly increased wound healing in Huh-7 cells
4. Overexpression of HK2, PKM2, and LDHA markedly increased Matrigel invasion capacity in Huh-7 cells. Conversely, knockdown of HK2, PKM2, and LDHA blocked Matrigel invasion capacity in HCCLM3 cells.

***Figure S2 related to Figure 2. lncRNA-SOX2OT is up-regulated in HCC of higher metastatic potential.***

1. lncRNA expression profile in 10 pairs of HCC tissue having different metastatic potential.
2. The effect of lncRNA- SOX2OT overexpression and knockdown. (## p<0.001)
3. lncRNA-SOX2OT overexpression promotes wound healing in Huh7 and Hep3B cells. Reciprocally, lncRNA-SOX2OT knockdown blocks wound healing in HCCLM3 and MHCC97-H cells. (## p<0.001)

***Figure S3 related to Figure 3. Effect of lncRNA-SOX2OT on in vivo tumor metastasis of HCC cells.***

1. The average tumor volume of HCCLM3 cells stably transfected with lncRNA-SOX2OT was much larger than tumor volume in the control group Conversely, the depletion of lncRNA-SOX2OT resulted in much smaller tumor volume. (* p<0.05)
2. lncRNA-SOX2OT overexpression increased lung metastases burden of HCCLM3 cells. Conversely, the depletion of lncRNA-SOX2OT blocked lung metastases.

***Figure S4 related to Figure 4. Effect of lncRNA-SOX2OT on in vivo tumor metastasis of HCC cells.***

1. No significant changes of oxygen consumption were observed after lncRNA-SOX2OT overexpression or knockdown.
2. No significant changes of ATP level were observed after lncRNA-SOX2OT overexpression or knockdown.
3. No significant correlations were observed between expression of lncRNA-SOX2OT and mRNA levels of HK1, HK2, LDHA and PFK.
4. No significant correlations were observed between expression of lncRNA-SOX2OT and protein levels of HK1, HK2, LDHA and PFK.
5. Immunofluorescence staining indicated that overexpression of lncRNA-SOX2OT could promote PKM2 protein level. Reciprocally, lncRNA-SOX2OT knockdown inhibit PKM2 protein level.

***Figure S5 related to Figure 5. miR-122-5p is a direct target of lncRNA-SOX2OT.***

1. miR-122-5p has one targeting site of PKM2.

***Figure S6 related to Figure 6. miR-122-5p mediates the role of lncRNA-SOX2OT in regulating PKM2, glucose metabolism and HCC cell metastasis.***

(A) The effect of miR-122-5p overexpression and knockdown. (## p<0.001)

(B) Ectopic expression of miR-122-5p blocks the effect of lncRNA-SOX2OT in promoting glycolysis and lactate production. Reciprocally, knockdown of endogenous miR-122-5p attenuate the effect. (## p<0.001)

(C) The promoting effect of lncRNA-SOX2OT on HCC cell invasion was antagonized by miR-122-5p overexpression. One the contrary, knockdown of miR-122-5p attenuated the inhibition effect of shRNA-lncRNA-SOX2OT on invasion of HCCLM3 and Hep3B cells.

***Figure S7. lncRNA-SOX2OT promotes HCC invasion through regulating EMT***

(A and B) Overexpression of lncRNA-SOX2OT, but not the mutant, reduced mRNA and protein level of E-cadherin and ZO-1 and increased N-cadherin and vimentin. Ectopic expression of miR-122-5p abolished the effect. (# p<0.01)

(C and D) Protein levels of E-cadherin, ZO-1, N-cadherin and vimentin after overexpression and knockdown of lncRNA-SOX2OT.

(E) Immunofluorescence staining revealed that overexpression of lncRNA-SOX2OT blocked increased E-cadherin and ZO-1 expression in the cell membrane, and induced N-cadherin and vimentin. Conversely, the depletion of lncRNA-SOX2OT induced an epithelial phenotype, upregulated E-cadherin and ZO-1, and downregulated N-cadherin and vimentin.

(F) The results of immunohistochemistry indicated that lncRNA-SOX2OT overexpression exhibited the inhibition of the typical EMT phenotype, including focal increment of the epithelial marker E-cadherin and concurrent loss of vimentin and N-cadherin.
