# Supplementary material for: lncRNA-SOX2OT promotes hepatocellular carcinoma invasion and metastasis through miR-122-5p-mediated activation of PKM2
Source: Oncogenesis. 2020 May 28;9(5):54. doi: 10.1038/s41389-020-0242-z (PMC7256049; doi:10.1038/s41389-020-0242-z)
Supplement: Supplementary file 3 — Supplementary Table 2 [file 41389_2020_242_MOESM3_ESM.docx]

**Supplementary Table 2. Primary antibodies for WB, IHC and IF**

| Protein | **S**pecificity | Company |
| --- | --- | --- |
| E-cadherin | Mouse | Abcam, ab231303 |
| N-cadherin | Mouse | Abcam, ab76057 |
| ZO-1 | Rabbit | Abcam, ab216880 |
| Vimentin | Rabbit | Abcam, ab92547 |
| ß-actin | Mouse | Abcam, ab8226 |
| N-cadherin | Mouse | BD, 610920 |
| ZO-1 | Rabbit | Invitrogen, 61-7300 |
| Vimentin | Rabbit | Cell Signaling, #5741 |
| ß-tubulin | Rabbit | Cell Signaling, #2128 |
| HK1 | Rabbit | Cell Signaling, # 2024 |
| HK2 | Rabbit | Cell Signaling, #2867 |
| PFK | Rabbit | Cell Signaling, #8337 |
| PKM2 | Rabbit | Cell Signaling, #4053 |
| LDHA | Rabbit | Cell Signaling, #3582 |
| DAPI | Rabbit | Cell Signaling, #4083 |
| Anti rat IgG | Goat | Invitrogen, Alexa Flour 594 |
| Anti rabbit IgG | Goat | Invitrogen, Alexa Flour 594 |
| Anti mouse IgG | Goat | Invitrogen, Alexa Flour 488 |
