# Supplementary figures and images for: lncRNA-SOX2OT promotes hepatocellular carcinoma invasion and metastasis through miR-122-5p-mediated activation of PKM2
Source: Oncogenesis. 2020 May 28;9(5):54. doi: 10.1038/s41389-020-0242-z (PMC7256049; doi:10.1038/s41389-020-0242-z)

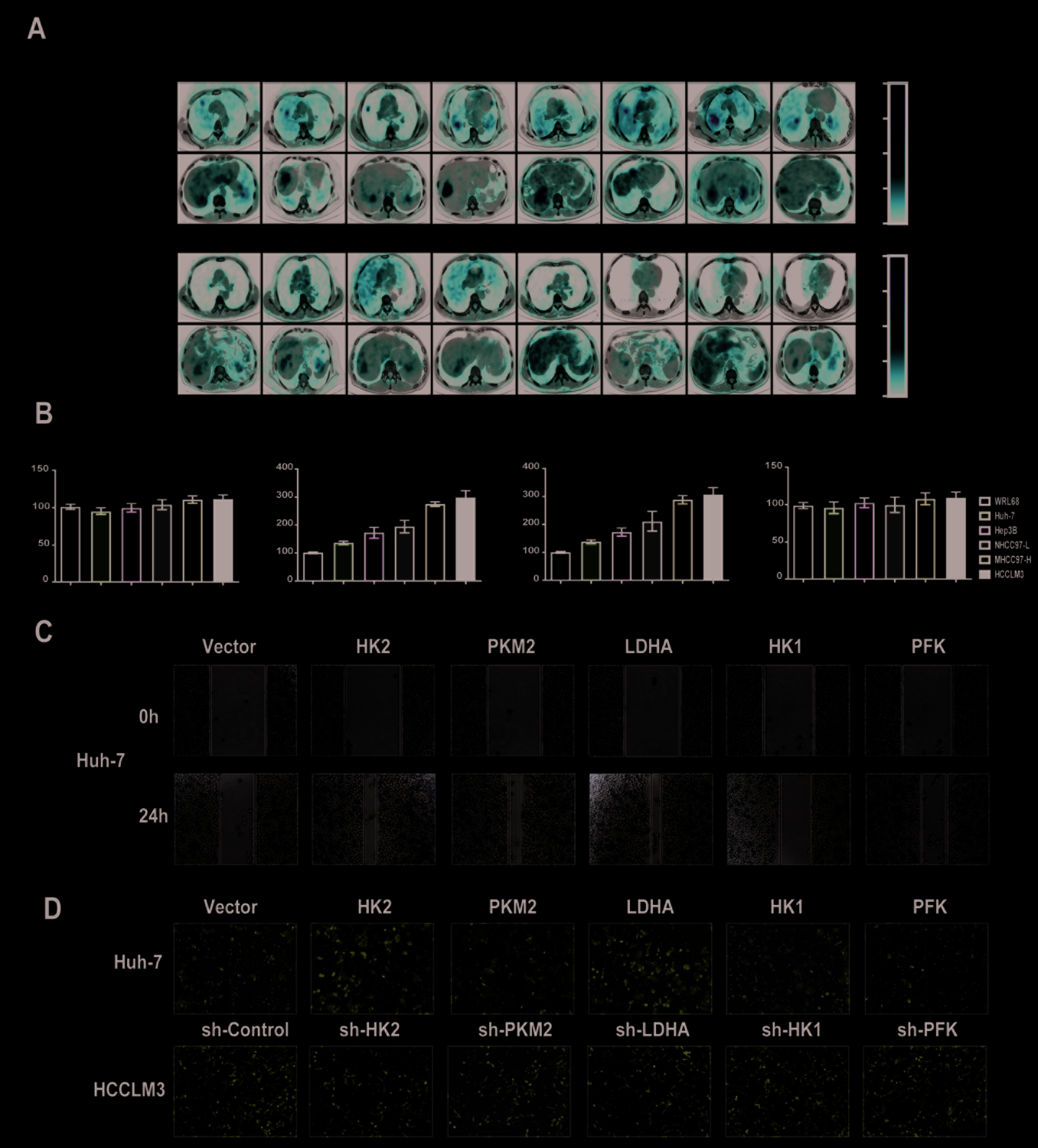

Supplement: Supplementary file 4 — Supplementary Figure 1 [file 41389_2020_242_MOESM4_ESM.tif]

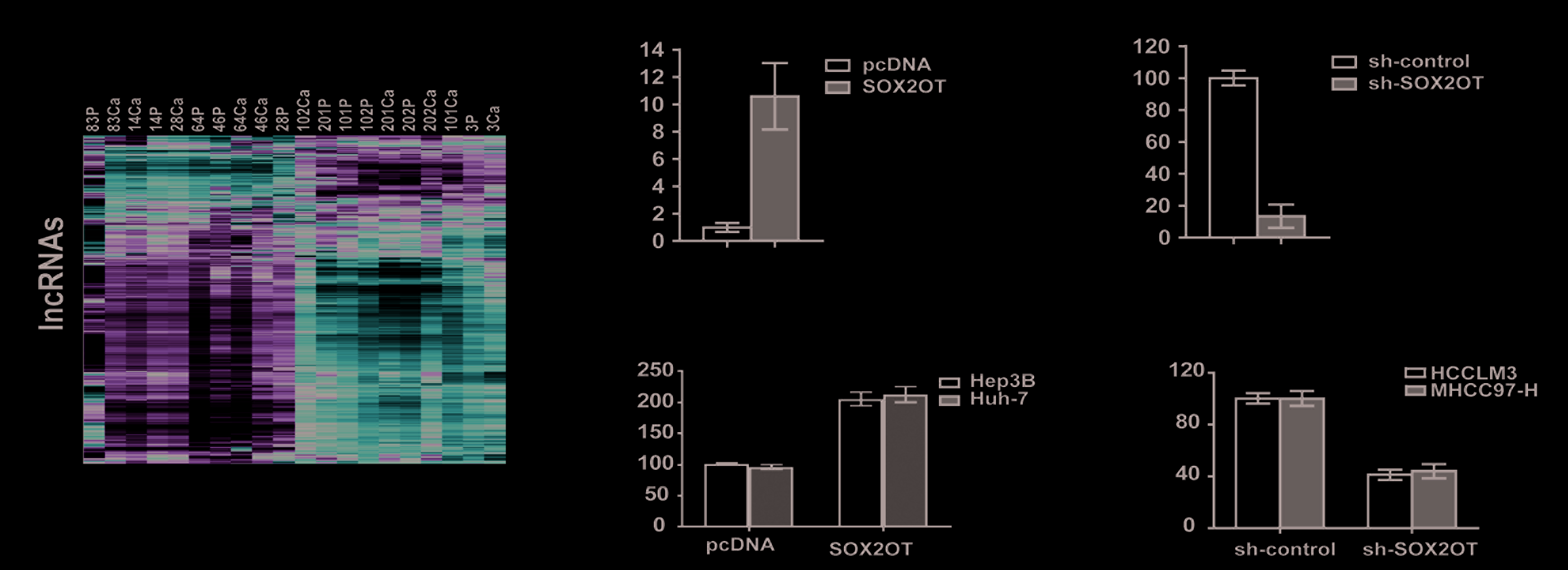

Supplement: Supplementary file 5 — Supplementary Figure 2 [file 41389_2020_242_MOESM5_ESM.tif]

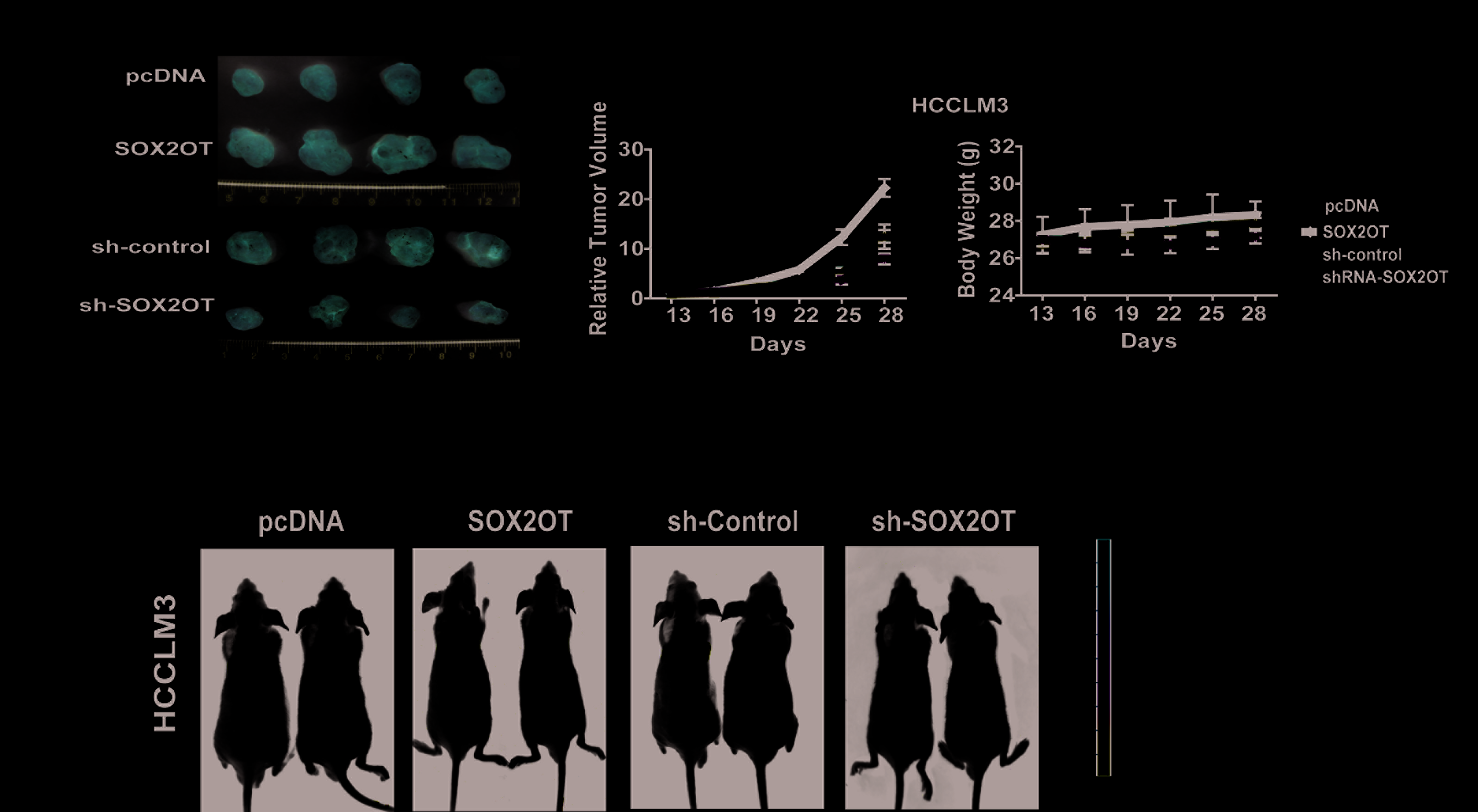

Supplement: Supplementary file 6 — Supplementary Figure 3 [file 41389_2020_242_MOESM6_ESM.tif]

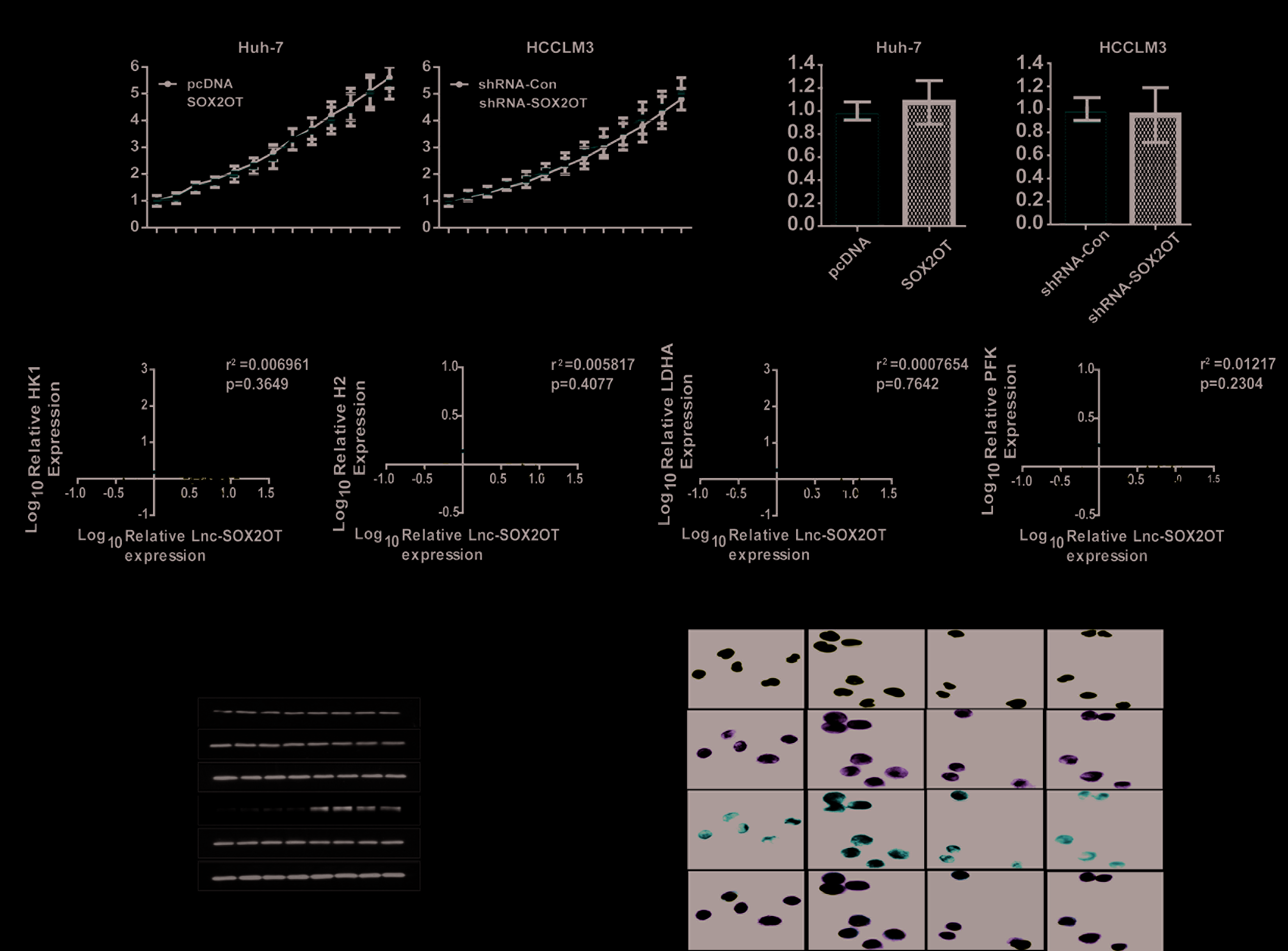

Supplement: Supplementary file 7 — Supplementary Figure 4 [file 41389_2020_242_MOESM7_ESM.tif]

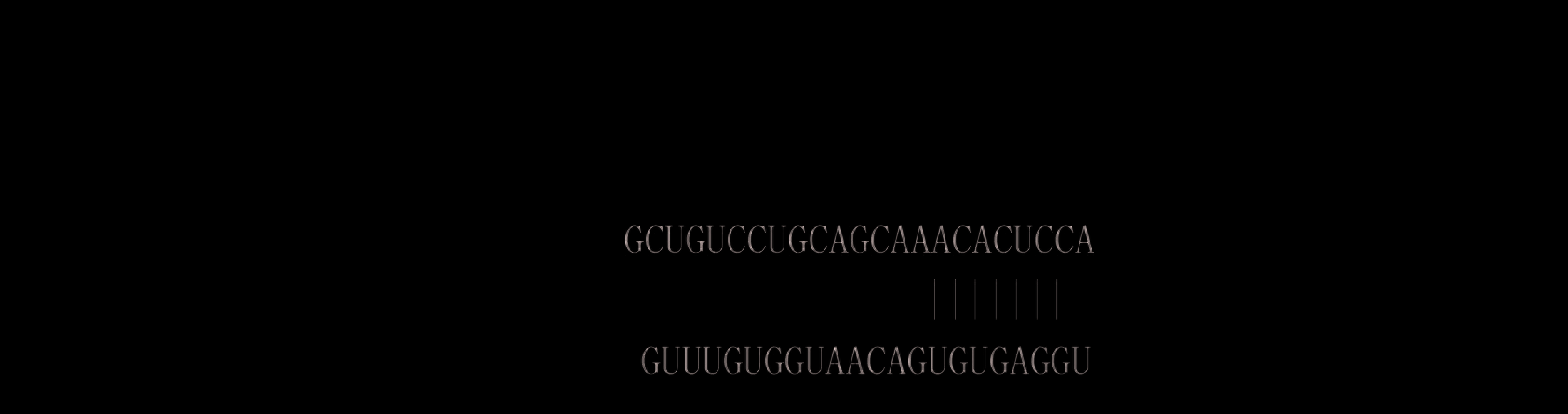

Supplement: Supplementary file 8 — Supplementary Figure 5 [file 41389_2020_242_MOESM8_ESM.tif]

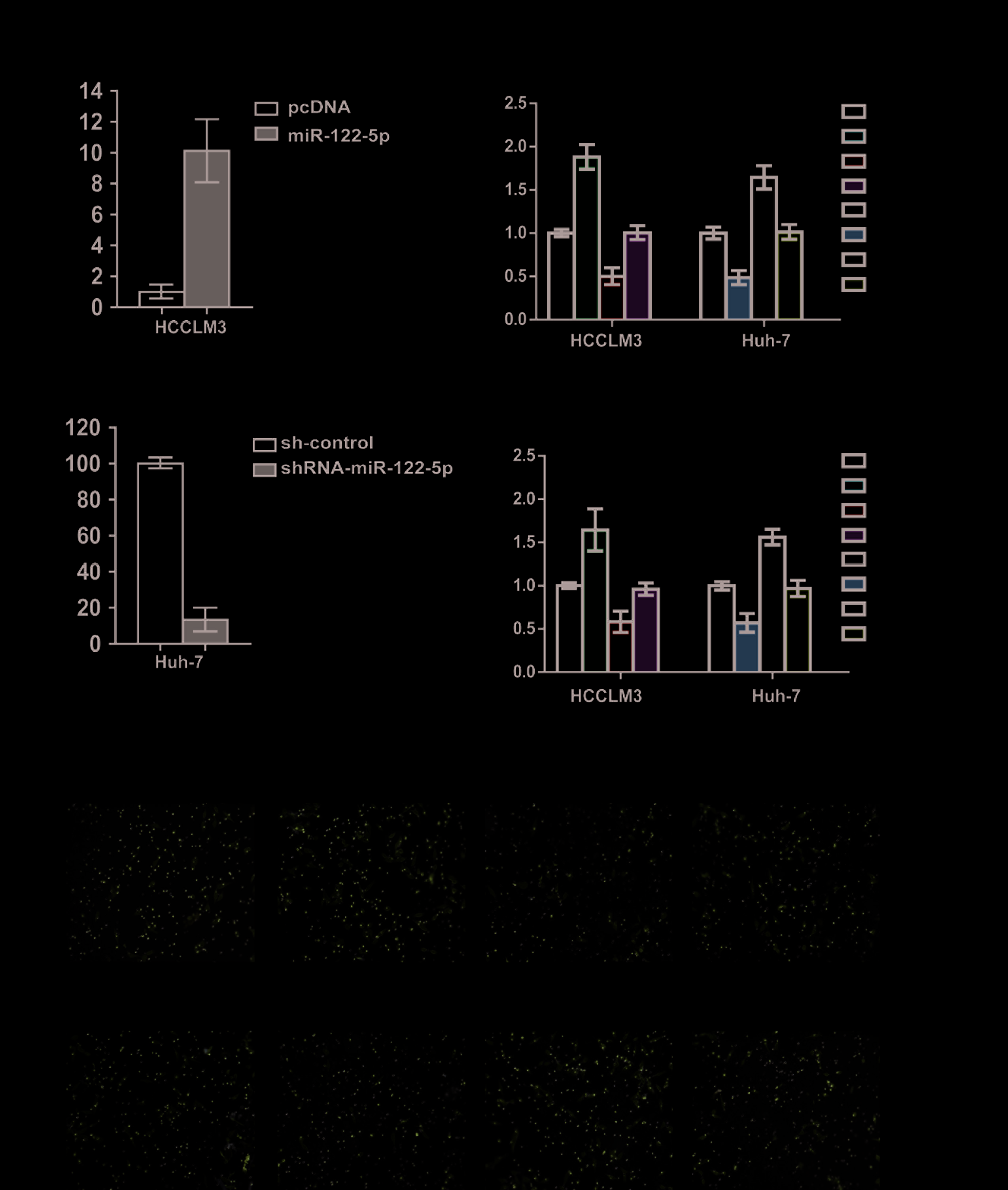

Supplement: Supplementary file 9 — Supplementary Figure 6 [file 41389_2020_242_MOESM9_ESM.tif]
